# Interleukin-6 blockade attenuates lung cancer tissue construction integrated by cancer stem cells

**DOI:** 10.1038/s41598-017-12017-y

**Published:** 2017-09-26

**Authors:** Hiroyuki Ogawa, Michiyo Koyanagi-Aoi, Kyoko Otani, Yoh Zen, Yoshimasa Maniwa, Takashi Aoi

**Affiliations:** 1 Division of Advanced Medical Science, Graduate School of Science, Technology and Innovation, Kobe University, Kobe, Japan; 2 Department of iPS Cell Applications, Graduate School of Medicine, Kobe University, Kobe, Japan; 3 Division of Thoracic Surgery, Graduate School of Medicine, Kobe University, Kobe, Japan; 4 Center for Human Resource Development for Regenerative Medicine, Kobe University Hospital, Kobe, Japan; 5 Department of Diagnostic Pathology, Graduate School of Medicine, Kobe University, Kobe, Japan

## Abstract

In the present study, we successfully generated lung cancer stem cell (CSC)-like cells by introducing a small set of transcription factors into a lung cancer cell line. In addition to properties that are conventionally referred to as CSC properties, the lung induced CSCs exhibited the ability to form lung cancer-like tissues *in vitro* with vascular cells and mesenchymal stem cells, which showed structures and immunohistological patterns that were similar to human lung cancer tissues. We named them “lung cancer organoids”. We found that interleukin-6 (IL-6), which was expressed in the lung induced CSCs, facilitates the formation of lung cancer organoids via the conversion of mesenchymal stem cells into alpha-smooth muscle actin (αSMA)-positive cells. Interestingly, the combination of anti-IL-6 antibody and cisplatin could destroy the lung cancer organoids, while cisplatin alone could not. Furthermore, IL-6 mRNA-positive cancer cells were found in clinical lung cancer samples. These results suggest that IL-6 could be a novel therapeutic target in lung cancer.

## Introduction

Cancer stem cells (CSCs) including lung CSCs are cells that can reconstitute cancer tissues and which are considered to be responsible for cancer progression, metastasis and therapeutic resistance, and which result in a poor prognosis^1,2^. The Biology of lung CSCs remains unclear, and elucidating the molecular mechanism underlying the behavior of lung CSCs could lead to a complete cure for lung cancer^2,3^. However, as CSCs comprise only a small amount of cancer tissues, sampling limitations remain a major obstacle in CSC research. To overcome this obstacle, we generated CSC-like cells from a colon cancer cell line by the ectopic expression of a small set of transcription factors^4^. The cells were capable of forming tumors that were similar—in both structure and immunohistological pattern—to human colon cancer tissues^4^. We considered that we could apply the technology of inducing CSC-like cells to other types of cancer and use the technology to develop novel cancer treatments^5^.

In this study, we established technologies to generate lung CSC-like cells from human lung cancer cell line A549 by introducing OCT3/4, SOX2 and KLF4, and to construct “lung cancer organoids” *in vitro* that mimicked human lung cancer tissues. Through the use of these technologies and the evaluation of clinical samples, we identified interleukin-6 as a novel potential therapeutic target for lung cancer stem cells.

## Results

### The induction of lung cancer stem-like cells by the ectopic expression of OCT3/4, SOX2 and KLF4 in a human lung adenocarcinoma cell line

#### i)Transduction of OCT3/4, SOX2 and KLF4 induced slow-growing and spherogenic cells

**Figure 1:**
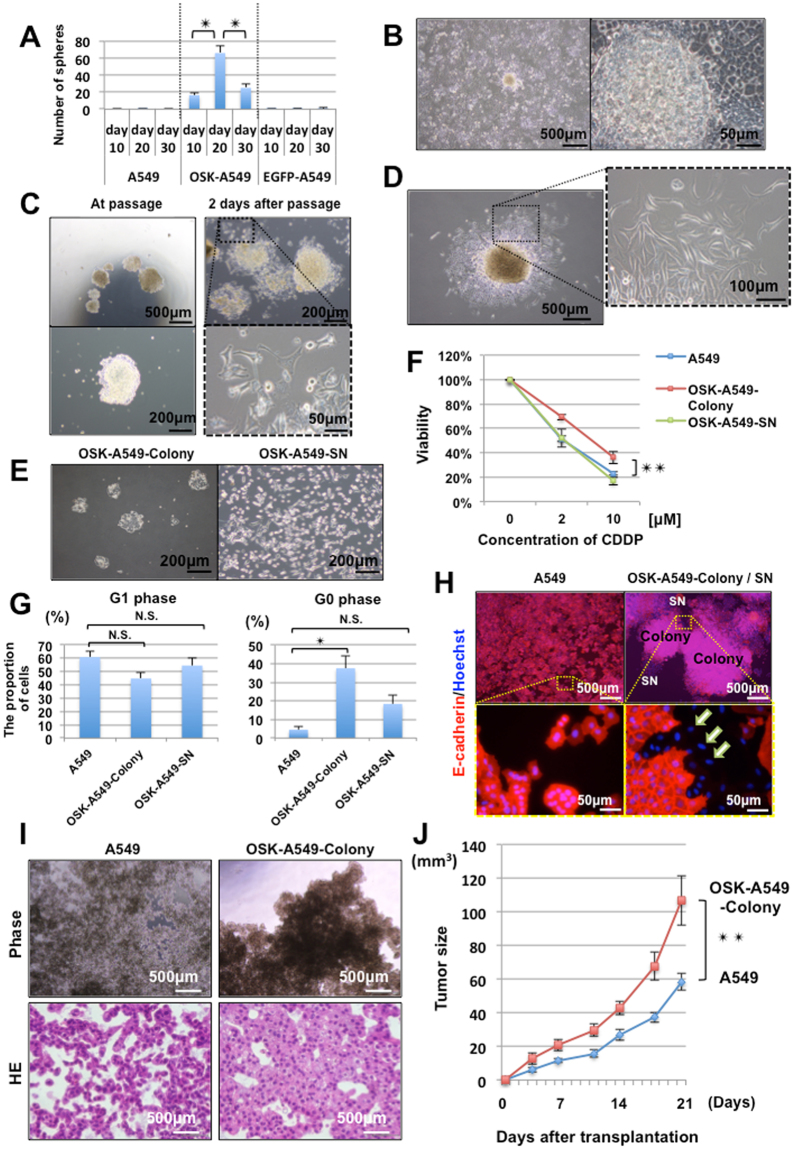
The induction of lung cancer stem-like cells and their characteristics. (**A**) A comparison of the sphere formation ability. (n = 3, *P < 0.05, Bonferroni test). (**B**) Dome-shaped colonies appeared in OSK-A549 cells at 10 to 15 days after the transduction of OSK. (**C**) Pictures of the colonies taken during passaging (left panels) and at 2 days after passaging (right panels). Spindle-shaped colonies cells appeared around the colonies after passaging. (**D**) The passaged colonies grew larger and gave rise to various cell phenotypes; most of the cells were spindle-shaped. (**E**) The cellular morphology of the OSK-A549-Colony cells (left panel), and OSK-A549-SN cells (right panel). After trypsinizing the OSK-A549-Colony cells for approximately 6 minutes, only the spindle-shaped cells around the colonies were detached; we collected them as supernatant cells (SN cells). (**F**) Chemoresistance among the A549, OSK-A549-Colony, and OSK-A549-SN cells following 3 days of cisplatin (0, 2, 10 µM) treatment. (n = 3, **P < 0.01; repeated measures ANOVA). (**G**) The cell cycle was analyzed by flow cytometry based on Ki67 and Hoechst staining. (n = 3, *P < 0.05; Dunnett’s test). (**H**) Immunocytochemistry of E-cadherin and Hoechst staining in the parental A549 and OSK-A549-Colony/SN cells. E-cadherin-negative cells were found around the OSK-A549-Colony cells (indicated as white arrows). (**I**) Phase contrast microscopy of the spheres (upper panels) and HE staining images (lower panels). Phase contrast microscopy of the OSK-A549-Colony cells showed obvious cellular aggregation, but not the parental A549 cells on the 24-well low attachment plate. (**J**) A comparison of the tumorigenicity *in vivo* between the parental A549 and OSK-A549-Colony cells (n = 10, **P < 0.01; repeated measures ANOVA).

We transduced OCT3/4, SOX2, and KLF4 (hereafter, OSK) or EGFP into a KRAS-mutated (G12S) human lung adenocarcinoma cell line (A549) using retrovirus vectors, then cultured the cells in 10% fetal bovine serum (FBS) containing Dulbecco’s modified Eagle’s medium (DMEM). Passaging was performed before the cells reached confluence. These OSK- or EGFP-transduced A549 cells were termed OSK-A549 cells or EGFP-A549 cells, respectively. At two weeks after transduction, the growth rate of OSK-A549 cells decreased in comparison to the parental A549 and EGFP-A549 cells (Figure S1A). To assess the sphere formation ability, which is considered to be a property of cancer stem cells, we cultured these cells on low attachment plates on days 10, 20, and 30 after transduction. The parental A549 cells and EGFP-A549 cells formed less than 3 spheres under this condition. In contrast, the number of spheres formed by the OSK-A549 cells was remarkably increased, especially on day 20 after transduction (Figs 1A, S1B).

#### ii)OSK-A549 cells contained subsets with distinct morphology and trypsin sensitivity

Dome-shaped colonies appeared only in the OSK-A549 cells from 10 to 15 days after transduction (Fig. 1B). The colonies were picked up and sub-cultured (Fig. 1C). During one week of continuous culture, these colonies grew larger and gave rise to spindle-shaped cells around themselves (Fig. 1D). We succeeded in collecting the colony cells and the spindle-shaped cells separately by using the different sensitivity of the cells to detachment by trypsin: the spindle-shaped cells detached earlier, while the colony cells detached later (Fig. 1E). We termed the colony cells, “OSK-A549-Colony cells” and the spindle-shaped mesenchyme-like cells, “OSK-A549-SN cells”.

#### iii)The induced OSK-A549-Colony cells exhibited chemo-resistance and a delayed cell cycle

Since cancer stem cells are thought to be particularly resistant to anticancer drugs^6,7^, we assessed the viability of the parental A549 cells, the OSK-A549-Colony cells and the OSK-A549-SN cells following exposure to cisplatin. In comparison to the other two cell lines, the OSK-A549-Colony cells were significantly more resistant to cisplatin (Fig. 1F). We next performed cell cycle analyses by flow cytometry based on Ki67 (a proliferation marker) and Hoechst 33342 staining because quiescence is an important mechanism of drug resistance in stem cells^6–8^. The proportion of Ki67-negative G0-phase cells in the OSK-A549-Colony cells was significantly increased in comparison to the parental A549 cells (Figs 1G, S1C). A cell cycle assay using redox dye consistently showed the increased proportion of G0/G1-phase cells in the OSK-A549-Colony cells (Figure S1D).

#### iv)OSK-A549-SN cells recapitulate the epithelial mesenchyme transition (EMT) and enhance the invasion ability

As cancer stem cells are considered to be responsible for cancer invasion^9,10^, we next investigated the EMT and the changes in the invasion ability of the induced cells. Immunocytochemistry of an epithelial marker (E-cadherin) showed the presence of E-cadherin-negative cells in OSK-A549-SN cells that had originated from OSK-A549-Colony cells; whereas, the parental A549 cells were almost all E-cadherin-positive (Fig. 1H). Flow cytometry to detect the expression of E-cadherin revealed that 38.3% of the OSK-A549-SN cells were E-cadherin-negative (Figure S1E).

A wound healing assay revealed that the migration ability of OSK-A549-SN cells was increased in comparison to the parental A549 cells (Figure S1F). The double-layered collagen gel hemisphere (DLCGH) method^11,12^ also showed that invasion ability of the OSK-A549-SN cells was significantly enhanced (Figure S1G,H). Moreover, observation with a HoloMonitor M4 (Digital holographic microscopy) revealed that OSK-A549-SN cells had the highest motility and migration ability among the three types of cells (Figure S2A,B).

#### v)The sphere formation ability of OSK-A549-Colony cells is enhanced

We further investigated whether the induced OSK-A549-Colony cells had sphere formation ability. Phase contrast microscopy showed obvious cellular aggregation in the OSK-A549-Colony cells, but not in the parental A549 cells (Fig. 1I). Hematoxylin and eosin (HE) staining revealed that the OSK-A549-Colony cells could form dense spheres with a glandular ductal structure, whereas the parental A549 cells could not (Fig. 1I). Alcian blue-PAS staining revealed the presence of polarized glandular ductal structures with mucin secretion in the OSK-A549-Colony cell-derived spheres (Figure S1I).

#### vi)The tumorigenicity of OSK-A549-Colony cells is enhanced

To examine the tumorigenicity *in vivo*, we subcutaneously transplanted 1 × 10^6^ parental A549 cells or OSK-A549-Colony cells into immunocompromised mice and measured the volume of the tumors that developed. The tumors derived from OSK-A549-colony cells were significantly larger than those derived from parental A549 cells (Fig. 1J).

Based on the above findings described in ***i)*** to ***vi)***, we considered the OSK-A549-Colony cells to be lung cancer stem-like cells.

### OSK-A549-Colony cells could form lung cancer organoids by co-culture with HUVECs and MSCs

**Figure 2:**
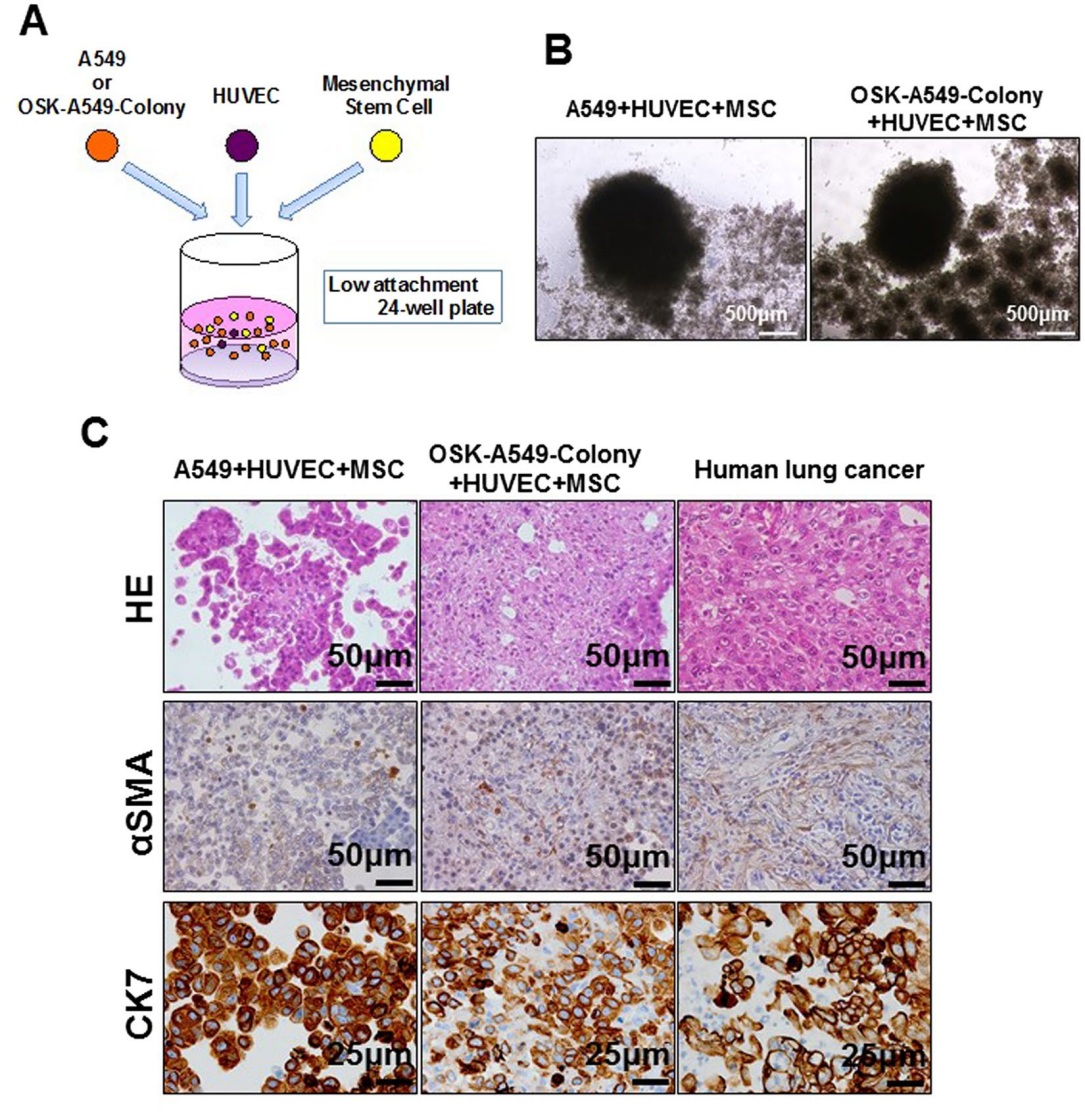
The construction of lung cancer organoids by induced OSK-A549-Colony cells. (**A**) A schematic illustration of the co-culture assay. (**B**) Phase contrast microscopy of the organoids made by co-culturing the parental A549 (left panel) or OSK-A549-Colony cells (right panel) with HUVECs and MSCs on low-attachment plates. (**C**) HE, α Smooth muscle actin (αSMA) and cytokeratin 7 (CK7) staining of the constructed organoids and a human lung cancer tissue (KRAS mutation-positive lung adenocarcinoma, papillary predominant pattern).

It is reported that liver organ buds can be generated by the co-culture of induced pluripotent stem cell-derived hepatocytes, human umbilical vein endothelial cells (HUVECs) and human mesenchymal stem cells (MSCs)^13^. The cellular interaction is considered to be important for this self-organization^14^. Not only normal tissues but also cancer growth has been thought to depend on dynamic interaction with adjacent stromal cells that compromise a niche^15^. We therefore investigated whether OSK-A549-Colony cells could induce self-organization by interacting with HUVECs and MSCs. We co-cultured the parental A549 or OSK-A549-Colony cells with HUVECs and MSCs on low attachment plates (Fig. 2A).

Phase contrast microscopy revealed no obvious differences between the parental A549 cell-derived and the OSK-A549-Colony cell-derived cellular aggregates (Fig. 2B). However, HE staining and immunostaining for alpha smooth muscle actin (αSMA) and Cytokeratin 7 (CK7) demonstrated that only the OSK-A549-Colony cells could form cohesive cell nests that were similar to human lung cancer tissues (Fig. 2C), and we called these nests “lung cancer organoids”. To visualize the cellular composition of the organoids, we transduced EGFP or mCherry into OSK-A549-colony cells or HUVECs, respectively and predicted the cellular viability by detecting the fluorescence intensity. HUVECs could survive in the OSK-A549-Colony cell-derived organoids but not in the parental A549 cell-derived organoids (Figure S3A,B), and an increased number of cluster of differentiation 31 (CD31; a marker of endothelial cells)-positive cells were found in the OSK-A549-Colony cell-derived organoids than in the parental A549 cell-derived organoids (Figure S3C).

In human lung cancer tissue specimens, Ki67-positive cells were more frequently detected on the outer side of the cancer cell clusters than on the inner side (Figure S3D). We found the peripheral-dominant distribution of Ki67-positive cells in the OSK-A549-Colony-derived organoids, but not in the parental A549-derived organoids (Figure S3D,E).

### The gene expression analysis of the parental A549, OSK-A549-Colony and OSK-A549-SN cells

**Figure 3:**
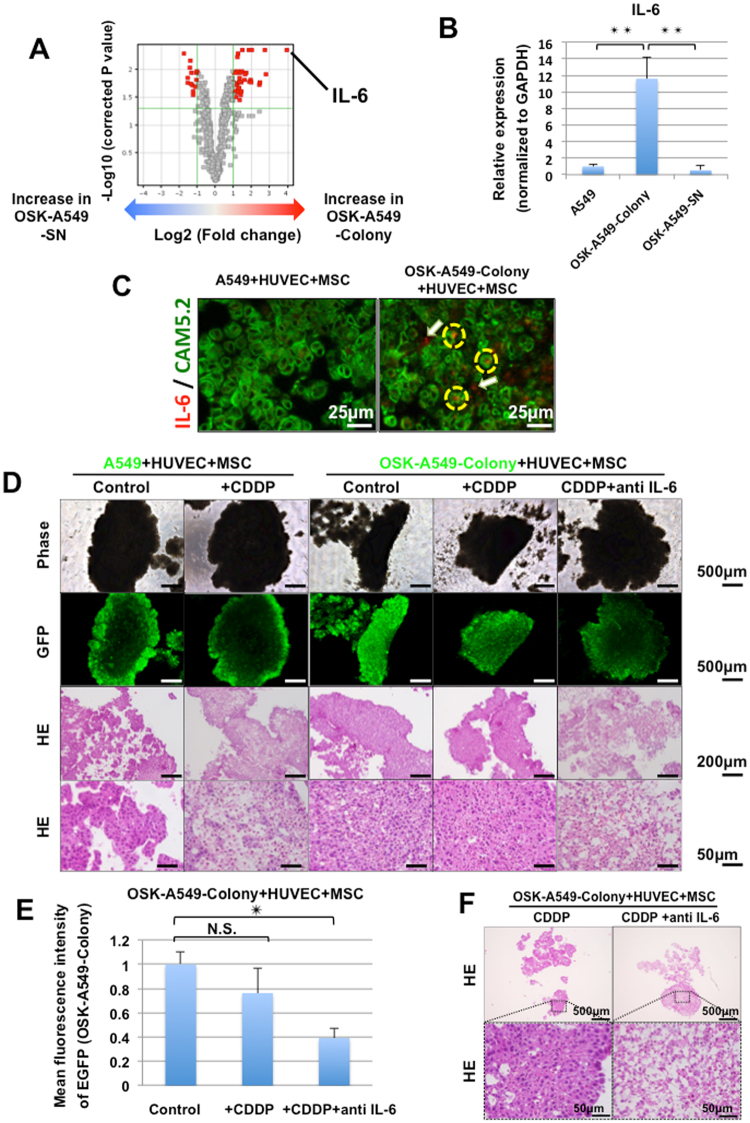
Interleukin-6 (IL-6) was a key factor in the acquisition of lung cancer stem cell-like properties. (**A**) A volcano plot showed that IL-6 was the gene that was most differentially expressed in the OSK-A549 Colony cells in comparison to the OSK-A549 SN cells. (**B**) Quantitative PCR showed that IL-6 was significantly upregulated in OSK-A549-Colony cells compared with the parental A549 and OSK-A549-SN cells (n = 3, **P < 0.01, Bonferroni test). (**C**) Double staining of IL-6 mRNA and CAM5.2 for the organoids derived from parental A549 and OSK-A549-Colony cells. ﻿Yellow dotted circles and white arrows indicate IL-6 mRNA detected in CAM5.2 positive and negative cells, respectively. ﻿(**D**) Phase contrast and GFP images of the constructed organoids with indicated drug treatments (upper two panels). The HE staining results are shown in the lower two panels. The GFP-positive cells were surviving A549 or OSK-A549-colony cells. (**E**) The mean fluorescence intensity was analyzed at 7 days after drug treatment using the Image J software program. (n = 3, *P < 0.05; Dunnett’s test). (**F**) HE staining of OSK-A549-Colony + HUVEC + MSC. Almost all of the OSK-A549-Colony cell-derived organoids were killed by co-culturing with 5 μM CDDP and IL-6 antibodies (1 μg/ml).

To clarify the molecular mechanisms that determined the properties of OSK-A549-Colony cells, we compared the gene expression patterns among the parental A549, OSK-A549-Colony, and OSK-A549-SN cells. At first, we compared the gene expression of the parental A549 cells and the OSK-A549-Colony cells. We found that 575 genes were differentially expressed in OSK-A549-Colony cells to a significant extent (P < 0.05; Fold change, >2.0). Among these genes, we compared the gene expression in the OSK-A549-Colony and OSK-A549-SN cells; the results are shown in a volcano plot (Fig. 3A). It showed that 54 of the genes that were differentially expressed between the OSK-A549-Colony cells and the OSK-A549-SN cells (P < 0.05; Fold change, >2.0; red plots in Fig. 3A). The expression of previously-reported candidate marker genes, such as CD133, CD44 and ABCG2, which are argued to be related to cancer stem cells, and the transduced genes OCT3/4, SOX2 and KLF4 were not significantly different among the parental A549, OSK-A549-Colony and OSK-A549-SN cells (Figure S4A,B). Although the expression of NANOG, a pluripotency marker, in OSK-A549 colony and OSK-A549-SN cells was significantly higher than in parental A549 cells, the absolute values of NANOG expression were small across all three kinds of cells.

Interleukin-6 (IL-6) was the gene that was most differentially expressed in OSK-A549-Colony cells in comparison to OSK-A549-SN cells (Fig. 3A). We performed a quantitative RT-PCR and confirmed that IL-6 was significantly upregulated in the OSK-A549-Colony cells in comparison to the other two cell types (Fig. 3B). Thus the present study focused on IL-6.

Interestingly, dual staining of IL-6 mRNA and CAM5.2 revealed that IL-6 mRNA was detected in both CAM5.2 positive and negative cells in the OSK-A549-Colony-derived organoids, but not in the parental A549-derived aggregates (Fig. 3C).

### IL-6 blockade enhanced the chemosensitivity of OSK-A549-Colony organoids

We next investigated the effects of IL-6 blockade on the chemosensitivity of the organoids by evaluating the fluorescence (EGFP) intensity of cancer cells and performing a histological examination of the organoids. The parental A549 cell-derived organoids were sensitive to 5 μM cisplatin (CDDP) (Fig. 3D). On the contrary, the OSK-A549-Colony cell-derived organoids were not (Fig. 3D). Notably, the evaluation of the mean fluorescence intensity of the cancer cells (Fig. 3D,E) and a histological examination (Fig. 3D,F) revealed that the OSK-A549-Colony cell-derived organoids were sensitive to the combination of cisplatin and anti-IL-6 antibodies (1 μg/ml).

### IL-6 plays an important role in the construction of lung cancer organoids

**Figure 4:**
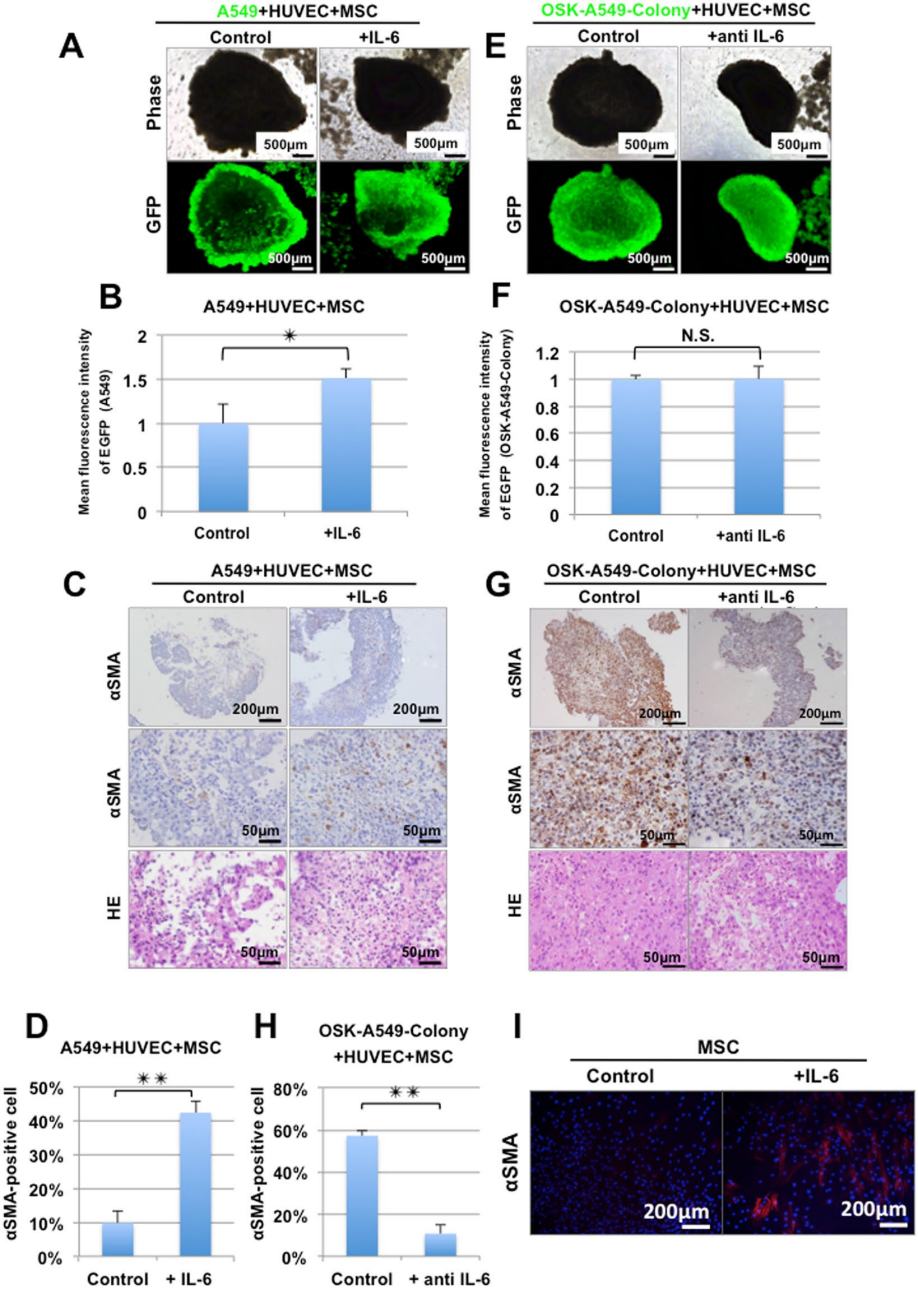
IL-6 plays an important role in the formation of lung cancer organoids. (**A**) Phase contrast and fluorescence images of the organoids consisting of EGFP-labeled parental A549 cells, HUVECs and MSCs. (**B**) The mean fluorescence intensity of GFP in the EGFP-labeled parental A549 cell-derived organoids. The addition of IL-6 significantly strengthened the mean fluorescence intensity (n = 3, **P < 0.01; two-tailed paired t-test). (**C**) HE and αSMA staining showed that the addition of IL-6 (10 ng/ml) increased the organoid forming ability of A549 + HUVEC + MSC by collaborating with the αSMA-positive cells. (**D**) The proportion of αSMA-positive cells in the parental A549 derived-organoids was increased by the addition of IL-6 (10 ng/ml) (n = 3, **P < 0.01; two-tailed paired t-test). (**E**) Phase contrast and fluorescence images of the organoids consisting of EGFP-labeled OSK-A549-colony cells, HUVECs and MSCs. (GFP shows cancer cells). (**F**) The mean fluorescence intensity of GFP in the EGFP-labeled OSK-A549-Colony cell-derived organoids. The addition of IL-6 antibodies did not attenuate the mean fluorescence intensity (n = 3, two-tailed paired t-test). (**G**) αSMA and HE staining showed that IL-6 signal blockade attenuated the organoid construction ability of OSK-A549-Colony + HUVEC + MSC, and that the proportion of αSMA-positive cells in the organoids was decreased. (**H**) The proportion of αSMA-positive cells in the organoids was decreased by the addition of IL-6 antibodies (n = 3, **P < 0.01; two-tailed paired t-test). (**I**) Immunocytochemistry for αSMA. Seven days treatment of IL-6 (10 ng/ml) could induce αSMA-positive cells from MSCs culture.

We evaluated the effects of IL-6 on the lung cancer organoid construction ability. The addition of IL-6 (10 ng/ml) strengthened the lung cancer organoid construction ability of the parental A549 cells (Fig. 4A,B). αSMA staining showed that the proportion of αSMA-positive cells in the parental A549 cell-derived organoid was significantly increased by the addition of IL-6 (Fig. 4C,D). In contrast, IL-6 blockade using anti-IL-6 antibodies (1 μg/ml) attenuated the proportion of αSMA-positive cells in the OSK-A549-Colony cell-derived organoids (Fig. 4G,H). Importantly, IL-6 blockade alone could not kill the lung cancer stem-like cells (Fig. 4E,F). HE and αSMA staining showed that the proportion of αSMA-positive cells in the OSK-A549-colony cell-derived organoids was significantly reduced by IL-6 blockade (Fig. 4G,H). Next, we confirmed that the addition of IL-6 could induce αSMA-positive cells from MSC cultures (Fig. 4I). These results suggest that lung cancer stem-like cells enhance the lung cancer organoid construction ability by inducing the conversion of MSCs into αSMA-positive cells.

### The identification of IL-6 mRNA-positive lung epithelial cells in the clinical samples

**Figure 5:**
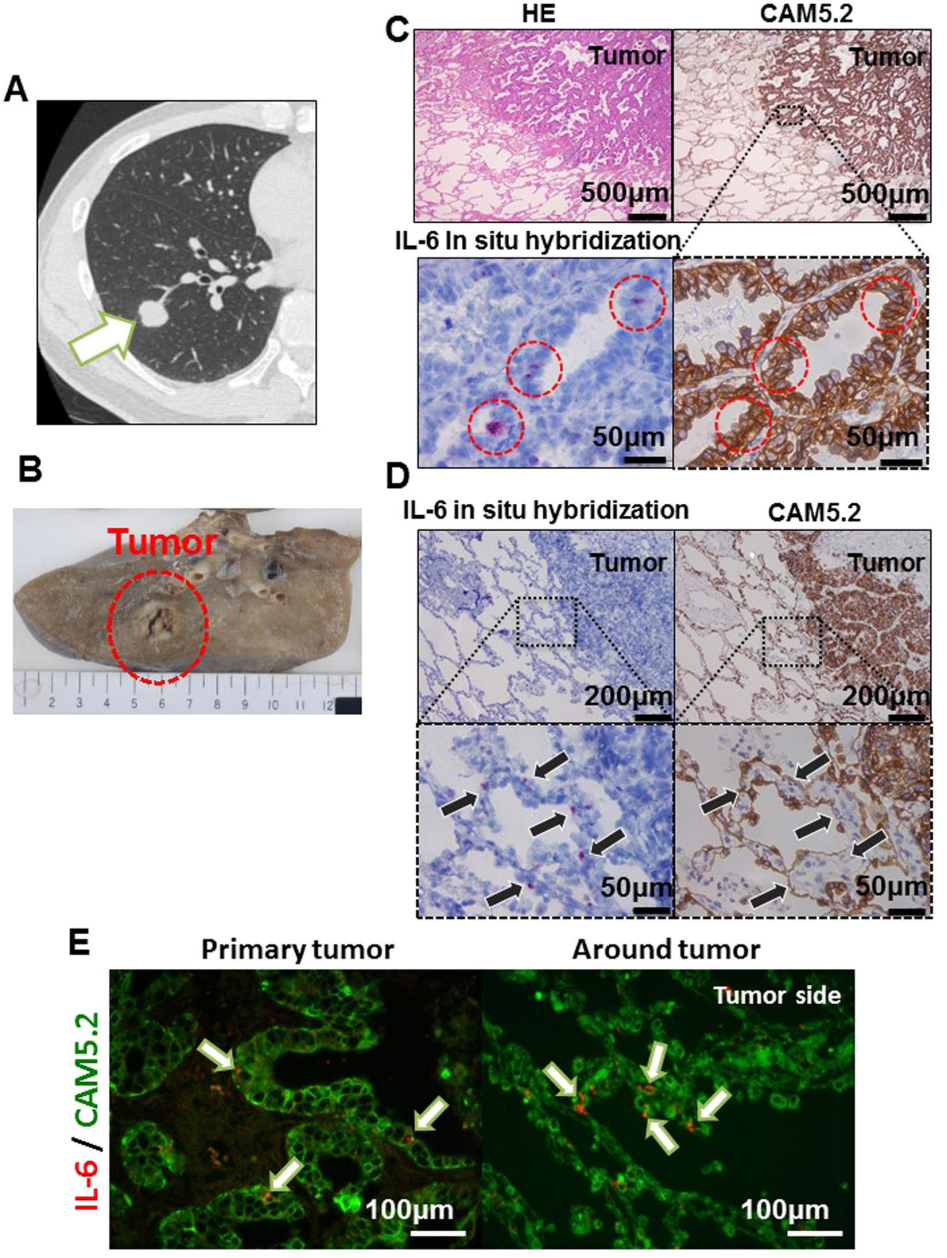
The IL-6 *in situ* hybridization of the clinical samples. A computed tomography (CT) image (**A**) and a picture of the surgical specimen (**B**) of a 52-year-old male patient with EGFR mutation-positive (Exon 19 deletion) lung adenocarcinoma (Case 4, lesion 5 in Table 1). (**C**,**D**) The *in situ* hybridization of IL-6 mRNA and HE and CAM5.2 staining of the cells in the tumor (**C**) and just around the tumor (**D**) in lung adenocarcinoma. IL-6 mRNA-positive lung cancer cells were detected in the clinical samples (red circle in **C**), and some normal lung epithelial cells located immediately around the tumor were also IL-6 mRNA-positive (arrow); however, the normal lung epithelial cells were mostly negative. (**E**) Double staining of IL-6 mRNA and CAM5.2 in the tumor cells and just around the tumor.

To evaluate whether IL-6-expressing lung cancer cells exist in human lung cancer tissues, we performed *in situ* hybridization for IL-6 using 10 lung adenocarcinoma tissue specimens from 8 patients of various backgrounds (Table 1). A representative CT image and an image of the surgical specimen are shown in Figs 5A,B, S5A,B. As a result, IL-6 mRNA-positive cells were detected in 8 of the 10 specimens. An immunohistochemical analysis to detect the CAM5.2 and E-cadherin epithelial markers in the same specimens showed that IL-6 mRNA-positive cells were not only found in mesenchymal cells (Figure S5C,D; arrowhead) but also in cancer cells (Figs 5C, S5C,D; red circle)—specifically, primary lung cancer tissues and a pulmonary metastasis lesion (Figure S5D). Moreover, IL-6 mRNA-positive cells were also found in non-cancer lung epithelial cells near a primary lung cancer lesion (Figs 5D, S5E; arrow). In order to exclude the possibility that the IL-6-expressing cells were intraepithelial lymphocytes or alveolar macrophages, we performed double staining of IL-6 mRNA and CAM5.2 in the same tissue sections and confirmed that double-positive cells definitely existed in human lung cancer tissues (Fig. 5E). In our evaluation of the 10 lesions in the 8 cases, IL-6 mRNA positivity seemed to have no obvious correlation with the background of any of the patients or any known driver gene mutations (Table 1).

**Table 1:** The results of IL-6 *in situ* hybridization for 10 lung cancer lesions.

Case	Lesion	Age	Sex	Smoking history	Brinkman index	p-Stage	TNM	Histological type	Mutation status	Primary or PM	*In situ* hybridization
1	1	69	F	Never	0	2B	T3N0M0	Invasive mucinous adenocarcinoma	ALK/EGFR/KRAS-negative	Primary	Negative
2	2	67	M	Ever	3360	3 A	T3N1M0	Papillary predominant	KRAS (G13C) positive	Primary	Positive
	3									PM	Positive
3	4	78	F	Ever	600	1 A	T1bN0M0	Papillary predominant	ALK-positive	Primary	Weakly positive
4	5	52	M	Never	0	2B	T3N0M0	Papillary predominant	EGFR-positive (Exon 19 deletion)	Primary	Positive
	6									PM	Positive
5	7	74	M	Ever	1600	1B	T2aN0M0	Papillary predominant	ALK/EGFR/KRAS-negative	Primary	Negative
6	8	65	F	Never	0	1 A	T1bN0M0	Papillary predominant	EGFR-positive (L858R)	Primary	Positive
7	9	60	M	Ever	800	1B	T2aN0M0	Invasive mucinous adenocarcinoma	KRAS (G12V)-positive	Primary	Positive
8	10	69	F	Ever	480	2B	T3N0M0	Acinar predominant	ALK/EGFR/KRAS-negative	Primary	Positive

## Discussion

Lung CSCs are considered to be responsible for the poor prognosis of patients with lung cancer due to therapeutic resistance and the possible development of recurrence or metastasis^1,16,17^. Thus, lung CSCs have been the subject of great interest. However, difficulty in obtaining proper research materials has hindered the elucidation of lung CSC biology and the development of novel therapies that target lung CSCs. In order to isolate lung CSCs from clinical specimens, several previous studies^17–21^ have attempted (but failed) to identify specific markers for lung CSCs via immunostaining. In the present study, we successfully generated lung CSC-like-cells by introducing defined factors into a KRAS-mutated lung cancer cell line (A549) and made lung cancer organoids that resembled *bona fide* human lung cancer tissues. Although patient-derived cancer organoid technologies in various types of cancers^22^ including lung cancer^23^ were recently reported, they do not provide isolated stem cell populations. We considered that the triad consisting of the lung CSC-like cells, the lung cancer non-stem cells differentiated from these cells and the organoids could be useful novel materials for lung CSC research. Indeed, we obtained new knowledge about lung CSCs by utilizing the triad in the current study.

Co-culturing with HUVECs and MSCs led the induced lung CSC-like cells—but not the parental A549 cells—to form organoids that were similar to human lung cancer tissues. Previously, Takebe *et al*. succeeded in making various organoids, which they termed, “organ buds”, by co-culturing the parenchymal cells of various tissues with HUVECs and MSCs^24^. It should be noted that in their report, fetal parenchymal cells could form organoids via self-organization, whereas adult parenchymal cells could not. These findings indicate that the type of parenchymal cell is a determinant of self-organization. Our current data suggest that only cancer stem cells, at least in lung cancer, can assemble parenchymal and non-parenchymal cells into cancer tissues. Sasai classified the basic processes of tissue self-organization into three categories: self-assembly, self-patterning and self-morphogenesis^14^. As far as we know, the present study is the first to demonstrate that stem cells proactively take the initiative in self-assembly.

We then revealed that IL-6, which was differentially expressed between the induced lung CSC-like cells and their derivatives, plays an important role in the formation of lung cancer organoids via the conversion of MSCs into αSMA-positive cells, which have been considered to exert large contractile forces in cancer tissues, thereby promoting cancer growth^25^. We also found that IL-6 mRNA-positive cancer cells actually exist in clinical lung cancer tissue samples. IL-6 is a cytokine with a wide range of physiological functions. It has been reported to be associated with chemoresistance^26–31^, progression^26,31–33^, the expansion of cancer stem cell-like cells and a poor prognosis in various types of cancer, including lung cancer^26,28,29,34–37^. These previous reports demonstrated the effects of extrinsic IL-6 on cancer cells, and showed that non-cancer cells (such as cancer-associated fibroblasts) in various cancers^38–40^, as well as MSC^41^, indeed secreted IL-6. However, in our present experiments, the parental A549 cells could not form lung cancer organoids—even when they were co-cultured with MSCs and HUVECs. This suggests that the production of IL-6 by lung CSCs and the consequent high IL-6 concentration just around the lung CSCs is important for the construction of lung cancer tissue. A few previous reports^30,31,33^ that showed the production of IL-6 by cultured cancer cells support our present findings which demonstrated the expression of IL-6 in the induced lung CSCs and a small number of the cancer cells in *bona fide* human lung cancer tissues.

The current results raise the hope that IL-6 signal blockade and conventional chemotherapy may be used as a combination therapy targeting both lung cancer stem cells and bulk cancer cells. Previously, the inhibition of IL-6 signaling has been reported to attenuate tumorigenicity—especially in KRAS-driven cancers^28,37,42,43^—and IL-6 knockdown in A549 cells was shown to strengthen the chemosensitivity to cisplatin *in vivo* ^29^, and our current data illuminate the potential of targeting IL-6 in the context of lung cancer stem cells. We should be concerned with both the survival of the individual lung cancer cells and with the interaction among the lung cancer stem and non-stem cells and non-cancer cells (such as cancer-associated fibroblasts) that constitute cancer tissues. Although xenografts have been the standard method^6^ for evaluating the effects of drugs on cancer tissues, there have been concerns about differences in the microenvironments of humans and recipient animals, both in terms of species differences in non-parenchymal cells and immunodeficiency^6^. The lung cancer organoids that were established in the present study provide complementary evidence to support the efficacy of IL-6-targeted therapy and strengthen the rationale for a clinical trial of the therapy. Moreover, that we found IL-6 mRNA-positive cancer cells in KRAS mutation-negative cases suggests the potential for IL-6-targeted therapy to be applied to various types of lung cancer. A monoclonal IL-6 receptor blocker (tocilizmab) has already been used in clinical practices for rheumatoid arthritis^44–46^ and Castleman disease^45,47^. Thus, it will be much easier to apply in the clinical treatment of lung cancer patients than a newly discovered drug.

In addition to IL-6, we might be able to uncover other factors that are important for the tissue reconstruction ability of lung cancer stem cells by utilizing our currently established methods. Interestingly, we observed that the HUVECs in the lung cancer organoids survived for longer than the HUVECs in the co-cultures of parental A549, MSCs and HUVECs. This suggests some encouragement from the lung CSC-like cells. A GO term analysis revealed that the genes associated with “blood vessel development” were upregulated in the lung CSC-like cells (Table S1). Further evaluation will be necessary to clarify the functions of these genes in the reconstruction of lung cancer tissue. Multicellular systems like the lung cancer organoids are considered to involve huge numbers of regulatory components; however, it is inferred that there should be robust interactions among a limited number of core control modules that play predominant roles in the critical dynamics of the complex system^48^. A strategy that includes the comparison of the molecular signature between lung CSC-like cells and non-stem cells and phenotype analyses in *in vitro* lung cancer organoid assays may help to elucidate the complex mechanisms involved in lung cancer tissue construction and may lead to novel therapies for lung cancer patients.

## Materials and Methods

### Cell lines and cell culture

The human lung adenocarcinoma cell line (A549) was purchased from the Riken Bioresource center (RCB0098). The cells were cultured in Dulbecco’s modified Eagle’s medium (DMEM) (Nacalai Tesque, Kyoto, Japan) containing 10% fetal bovine serum (FBS) (Life Technologies), penicillin (100 Units/ml) and streptomycin (100 mg/ml) (Life Technologies) at 37 °C in 5% CO_2_; cells from an early passage were used for the experiments. HUVECs and Human MSCs were purchased from Lonza. HUVECs were maintained in endothelial growth medium (Lonza) at 37 °C in 5% CO_2_. Human MSCs were maintained in MSC growth medium (Lonza) at 37 °C in 5% CO_2_ and were used before Passage 5. We confirmed all of the cell lines were free of mycoplasma contamination by using a mycoplasma detection kit (Takara).

### Retroviral transduction

For retroviral transduction we modified previously described methods^4^, and used pMXs-OCT3/4, pMXs-SOX2 and pMXs-KLF4 vectors obtained from Addgene. PLAT-A packaging cells were plated at 1.2 × 10^6^ cells per 6 cm dish, and were incubated overnight. On the following day, the cells were transfected with pMXs vectors using the Fugene HD transfection reagent (Promega). pMXs-EGFP was used as a transfection control. At 24 h after transfection, the medium was replaced with new medium, which was collected (as the virus-containing supernatant) after another 24 h. The virus-containing supernatants were filtered through a 0.45-μm pore filter and supplemented with 4 μg/ml polybrene (Nacalai Tesque). Equal amounts of supernatants containing each of the retroviruses were mixed, transferred to the cancer cell line or HUVEC dish that was prepared the previous day, and incubated overnight. At another 24 h after infection, the virus-containing medium was replaced with fresh medium without retroviruses.

### The method of culturing OSK-A549-Colony and OSK-A549-SN cells

OCT3/4-, SOX2- and KLF4-transduced A549 cells were maintained in DMEM containing 10% FBS and 0.5% penicillin and streptomycin at 37 °C in 5% CO_2_. Colonies appeared in the OSK-A549 cells from 10 to 15 days. We then trypsinized them for 6 minutes with 0.25% trypsin and aspirated the trypsin. After removing the trypsin, the remaining cells were washed with PBS twice. The colony cells remained on the dish, and were picked up, gently pipetted 5 to 7 times and transferred to a new dish. Approximately 5 colonies in a 60 mm dish was sufficient for passaging. From 7 to 10 days after passaging, larger colonies appeared and spindle-shaped cells were found around the colonies. In order to passage colonies, they were trypsinized for 6 minutes with 0.25% trypsin. The dissociated cells in the supernatant were then collected and as OSK-A549-SN cells. After collecting the OSK-A549K-SN cells, the remaining cells were washed with PBS twice and the colonies were picked up, pipetted 5 to 7 times, and then transferred to a new dish.

### The sphere formation assay

The cells were transferred to a low-attachment 24-well flat plate (Prime surface, Sumitomo) in serum-free DMEM containing 10 ng/ml bFGF (Wako), 10 μg/ml human insulin (CSTI), 100 μg/ml human transferrin (Roche) and 100 μg/ml BSA (Nacalai Tesque) and incubated at 37 °C in a 5% CO_2_ incubator. In Fig. 1A, 1.0 × 10^4^ cells were seeded per well and cultured for 10 days. The spheres that were larger than 100 μm were counted. In Fig. 1I, 4.0 × 10^5^ cells were seeded per well and cultured for 6 days.

### Tumorigenicity *in vivo*

A total of 1.0 × 10^6^ cells in 100 μl of serum-free PBS were injected subcutaneously into both dorsal flanks of 8-week-old male BALB/c nude mice. The tumor volume was calculated by the formula 0.5 × L × W^2^ (L: length, W: width). The experiments were reviewed, approved by the Animal Welfare Committee, Kitayama Labes Co., Ltd. (Nagano, Japan) (Permit Number: IBC53-016), and conducted in accordance with institutional guidelines. All efforts were made to minimize suffering.

### Co-culturing with HUVECs and MSCs

To generate spheres *in vitro*, we resuspended ~1.0 × 10^6^ the parental A549 or ~1.0 × 10^6^ OSK-A549-colony cells, ~3.0 × 10^5^ HUVECs, and ~3.0 × 10^5^ MSCs were resuspended in sphere forming medium and seeded on a low-attachment 24-well flat plate (Prime surface Sumitomo). We adopted the following specific ratios with reference to a previous report^24^. A549 or OSK-A549-Colony cells: HUVEC: MSC = 10:1:4 ~ 5:4:4. During 6 to 12 days of culture, the self-organized spheres were photographed using a BZ8000 microscope (Keyence) and were pathologically analyzed. To assess the chemosensitivity of the spheres and the IL-6 function of the forming spheres, the spheres were co-cultured with or without 5 μM of cisplatin, 1 μg/ml of anti-IL-6 antibody (R & D, MAB206) and 10 ng/ml of IL-6 protein from the first day of this assay. The photographed pictures were then analyzed to calculate fluorescence intensity using the ImageJ software program.

### The histological and immunohistochemical analyses of the spheres and the clinical lung cancer specimens

The spheres and the clinical lung cancer specimens were embedded in paraffin blocks, and cut into 5-μm-thick sections. The sections were deparaffinized and stained with Hematoxylin and Eosin (HE), anti-αSMA mouse monoclonal antibody (Clone: 1A4, dilution 1:50, Dako), anti-human cytokeratin 7 (CK7) mouse monoclonal antibody (Clone: OV-TL 12/30, dilution 1:50, Dako), anti-CD31 mouse monoclonal antibody (Clone: JC70A, dilution 1:50, Dako) and anti-Ki-67 mouse monoclonal antibody (Clone: MIB-1, dilution 1:50, Dako). Immunohistochemistry was performed using a Benchmark XT (Roche) autostainer with the XT UltraView Universal DAB Detection Kit (Ventana Medical Systems, Inc.). We calculated the proportion of Ki67- and αSMA-positive cells by counting the immunohistochemically-positive cells and the total number of nuclei in three microscopic high power fields (HPF).

### RNA isolation and the quantitative reverse-transcriptase polymerase chain reaction

Total RNA was extracted from the parental A549 cells, OSK-A549-Colony cells, and OSK-A549-SN cells with TRizol (Life Technologies) and 500 ng of total RNA was reverse transcribed using a PrimeScript™ II 1st strand cDNA Synthesis Kit (Takara) according to the manufacturer’s protocol. A quantitative reverse-transcriptase polymerase chain reaction (qRT-PCR) was performed with the SYBR® Premix Ex Taq™ II (Tli RNaseH Plus) (Takara). GAPDH primers: forward, agccacatcgctcagacac; reverse, gcccaatacgaccaaatcc. IL-6 primers: forward, tacccccaggagaagattcc; reverse, ttttctgccagtgcctcttt. A LightCycler® 480 Real time PCR system (Roche) was used to quantify the absolute gene expression according to the standard curve method.

### The microarray and data analyses

RNAs from the parental A549 cells, OSK-A549-Colony cells, and OSK-A549-SN cells were obtained from three independent retroviral transduction experiments. Gene expression profiling was carried out using the SuperPrint G3 Human Gene Expression 8 × 60K v2 Microarray (Agilent Technologies) according to the manufacturer’s protocol. The data were analyzed using the GeneSpring GX 13.0 software program (Agilent Technologies). Control probes were removed and only the “detected” probes that were present in at least one of the analyzed samples were used for the further analysis. The number of probes used in the analysis was 30886 (the parental A549 vs A549-OSK-colony cells). Volcano plots were generated and a GO analysis was performed using the Gene Spring GX13.0 software program.

### Immunocytochemistry

For immunocytochemistry, the cultured cells, which were fixed with 4% paraformaldehyde, were stained with human E-cadherin antibody (R & D, dilution 1:100. AF 648) and Monoclonal Mouse Anti-Human Smooth Muscle Actin (Dako, Clone 1A4, dilution 1:500. M0851) and counterstained with Hoechst33342 (Life Technologies) to identify all of the nuclei.

### Flow cytometry

To analyze the expression of E-cadherin, the cells were re-suspended with PBS containing 2% FBS and mixed with 4% paraformaldehyde. The paraformaldehyde-fixed cells were permeabilized with 0.2% Triton-X (Sigma), and stained with E-Cadherin (24E10) Rabbit mAb (Alexa Fluor® 488 Conjugate) (CST 3199S). Rabbit IgG Isotype Control (Alexa Fluor® 488 Conjugate) (CST 4340S) staining was used as a control. These samples were analyzed using a FACS Aria III instrument (BD).

A cell cycle analysis using Ki67/Hoechst co-staining was performed according to a previously reported protocol^49^. Briefly, paraformaldehyde-fixed cells were permeabilized with 0.2% Triton-X (Sigma), and the cells were stained with PE Mouse Anti-Human Ki67 (1:50, BD, 556027) overnight in Permwash buffer (BD). PE mouse IgG1, k Isotype Control (1:50, BD, 556027) staining was used as a control. The cells were then stained for 5 min at RT with Hoechst 33342 (1:10,000; Life Technologies). The cells were analyzed by flow cytometry (FACS Aria III).

### The cell-cycle assay

A total of 2.0 × 10^4^ parental A549 and OSK-A549-Colony cells were plated on a 24-well plate and incubated for 24 hours. The cell-cycle was analyzed using a Cell-Clock Mammalian Cell Cycle Assay Kit (Biocolor life science assays) according to the manufacturer’s protocol.

### The wound healing assays

The parental A549 and OSK-A549-SN cells were seeded in 35 mm cell culture dishes. At 100% confluence, the cell layers were scratched using a plastic pipette tip. The wound healing of each cell was photographed at 30 hours.

### The double-layered collagen gel hemisphere (DL-CGH) assay

As previously reported^12^, acid-soluble collagen 1 (Nitta Gelatin Inc., Osaka, Japan), 10-fold-concentrated DMEM medium, and reconstruction buffer (2.2 g NaHCO3 + 4.77 g HEPES in 100 ml of 0.05-N NaOH) were mixed at a volume ratio of 8:1:1. Five microliters of mixture containing 3.0 × 10^4^ cells was then dropped onto a plastic dish. After 15 min of incubation at room temperature, a second 30-μl drop of collagen was placed directly on top of the first gel drop, encapsulating it completely. After 1 h of incubation at room temperature, the gel hemisphere was then submerged in medium and incubated at 37 °C for 2 weeks in a 5% CO_2_ incubator. The gel hemisphere was observed using a BZ8000 microscope (Keyence), and the invasion area was calculated using the ImageJ software program.

### The cisplatin sensitivity test

A total of 1.0 × 10^5^ of the parental A549, OSK-A549-Colony, and OSK-A549-SN cells were seeded in each well of 6-well plates and co-cultured with 2 μM or 10 μM of cisplatin (WAKO) for 3 days. The number of surviving cells was counted and the survival rate was calculated.

### Digital holographic microscopy

Twenty 3D holographic cell images were captured using a HoloMonitor M4 (Phase Holographic Imaging) with a 20x magnification objective and a low-power 635nm diode laser. Images were converted from wavelength interactions to cellular representations by a computer algorithm (HstudioM4, Phase Holographic Imaging) and the positional information, including the X and Y positions were recorded each 5 minutes. For the motility and migration analysis, we randomly selected 8 cells per HPF.

### RNA *in situ* hybridization

RNA *in situ* hybridization for human IL-6 mRNA was performed using an RNA scope Multiplex Fluorescent Reagent Kit (Advanced Cell Diagnostics, Inc. 320850) in accordance with the manufacturer’s instructions. In brief, 5-μm-thick formalin-fixed, paraffin-embedded tissue sections were deparaffinized and pre-treated with heat and protease before hybridization with the IL-6 oligonucleotide probes (425161-C1). Preamplifier, amplifier and alkaline-phosphatase-labeled oligonucleotides were then hybridized sequentially, coupled with a fluorescent conjugate. The RNA integrity quality was controlled with an RNAscope^®^ LS-positive Control Probe - Hs-PPIB (Advanced Cell Diagnostics, Inc. 313907), and for the background with an RNAscope^®^ -negative Control Probe-DapB (Advanced Cell Diagnostics, Inc. 310043). The specific RNA staining signal for IL-6 was identified as red.

For double staining of IL-6 mRNA and cytokeratin protein, after *in situ* hybridization for IL-6 mRNA, the sections were autoclaved in Tris buffer (pH 8.0; 2.5 min) for antigen retrieval and incubated for 60 min with a monoclonal antibody against human cytokeratins (BD, clone CAM5.2, dilution 1:10. AF 349205). The reaction product was visualized with Alexa Fluor® fluorescent goat anti-mouse IgG antibodies (1:500, Abcam, Cambridge, UK).

### Lung adenocarcinoma clinical samples

Patients with lung adenocarcinoma who underwent surgical resection in the department of thoracic surgery in Kobe University Hospital were randomly selected. The medical records were reviewed. The mutation status had already been clinically confirmed by a PCR (for EGFR mutations), fluorescence *in situ* hybridization (FISH) and immunohistochemistry (for EML4-ALK rearrangement), and the Scorpion-ARMS method (for KRAS mutations). The institutional review board of Kobe University Hospital approved the use of the tumor specimens (No. 160073). Written informed consent was obtained from all the patients.

### Statistical analysis

All data were analyzed using the JMP software program, ver. 9.0.0 (SAS Institute, Cary, NC, USA). The data values were presented as the mean ± standard error of the mean (SEM) of three independent experiments. Bonferroni’s test was used for the analyses of the data in Figs 1A and 3B. A repeated measures analysis of variance was used for the analysis of the data in Fig. 1F, 1J and S3B. Dunnett’s test was used for multiple comparisons in Figs 1G, 3E, S1H and S4. The differences in the mean values between two groups were analyzed using the two-tailed paired *t*-test in Fig. 4B, D, F, H and Fig. S3E. The differences were considered to be statistically significant for P-values < 0.05 (*) and <0.01 (**).

All methods were performed in accordance with the relevant guidelines and regulations including Declaration of Helsinki and Ethical Guidelines for Medical and Health Research Involving Human Subjects.

### Data Availability

The microarray data were deposited in the NCBI Gene Expression Omnibus under accession number GSE89229.

## Electronic supplementary material

Supplementary infornation
