# Supplementary material for: Interleukin-6 blockade attenuates lung cancer tissue construction integrated by cancer stem cells
Source: Sci Rep. 2017 Sep 26;7:12317. doi: 10.1038/s41598-017-12017-y (PMC5615065; doi:10.1038/s41598-017-12017-y)
Supplement: Supplementary file 1 — Supplementary infornation [file 41598_2017_12017_MOESM1_ESM.pdf]

Supplementary data.

Title: Interleukin-6 blockade attenuates lung cancer tissue construction integrated by cancer stem cells.

Hiroyuki Ogawa[1,2,3], Michiyo Koyanagi-Aoi[1,2,4], Kyoko Otani[5], Yoh Zen[5], Yoshimasa Maniwa[3], Takashi Aoi[1,2,4]

[1] Division of Advanced Medical Science, Graduate School of Science, Technology and Innovation, Kobe University.

[2] Department of iPS Cell Applications, Graduate School of Medicine, Kobe University

[3] Division of Thoracic Surgery, Graduate School of Medicine, Kobe University.

[4] Center for Human Resource Development for Regenerative Medicine, Kobe University Hospital.

[5] Department of Diagnostic Pathology, Graduate School of Medicine, Kobe University.

Figure S1 Phenotypes of the parental A549, OSK-A549, OSK-A549-Colony and OSK-A549-SN cells. A) The proliferation ability of OSK-A549 cells was decreased in comparison to the parental A549 and EGFP-A549 cells. B) Phase contrast microscopy of the spheres. Only OSK-A549 cells formed large numbers of spheres which were larger than 100 μm in size (the arrows indicate representative spheres). C) The cell cycle was analyzed by flow cytometry based on anti-Ki67-PE and Hoechst33342 staining. D) The results of a cell cycle assay using a cell-clock mammalian cell cycle assay kit. The yellow, green, and dark blue cells represent the G0/G1-phase, the S-phase, late G2- and M-phase cells, respectively. E) The analysis of the expression of E-cadherin by flow cytometry. F) The comparison of the migration ability by a wound healing assay. Cellular images taken during scratching (upper panels) and 30 hours later (lower panels) are shown. G) Cellular images of the three cell lines obtained using the DLCGH method. H) The invasion area in DLCGH was analyzed using the ImageJ software program (n=3, $^{**}P<0.01$; Dunnett's test). I) Alcian blue-PAS staining of the spheres of the parental A549 and OSK-A549-Colony cells.

Figure S2. The analysis of cellular motility and migration using a HolomonitorM4. A) Eight cells were selected and color-coded at the beginning of the analysis and were tracked for 5 hours (upper panels). Spatial movement graphs for selected cells (the starting position was the center) are shown in lower panels. B) Cellular movement was categorized by

non-directional cellular motility and directional migration velocity. The cellular motility speed of each cell line is shown in the upper panels and the cell migration velocity is shown in the lower panels. Both were increased in the OSK-A549-SN cells.

Figure S3. Co-culture of cancer cells, human umbilical vein endothelial cells (HUVECs) and mesenchymal stem cells (MSCs). A) Phase contrast (upper panels), red fluorescence (middle panels) and merged images (lower panels) of the co-cultured organoids consisting of EGFP-labeled cancer cells, mCherry-labeled HUVECs and MSCs. B) The mean fluorescence intensity of mCherry in the organoids shown in A. (n=3, *P<0.05; repeated measures ANOVA). C) The expression of CD31 was analyzed by immunohistochemistry to detect HUVEC in the organoids. D) Ki67 staining of the parental A549, OSK-A549-Colony cell-derived organoids and human lung cancer specimens. E) The proportion of Ki67-positive cells was decreased in the center of the OSK-A549-Colony cell-derived organoids. The cells located in the inner and outer halves of the spheres were counted as inner and outer cells, respectively (n=3, *P<0.05; two-tailed paired *t*-test.).

Figure S4. A gene expression analysis of the parental A549, OSK-A549-Colony and OSK-A549-SN cells by microarray. A) A comparison of the expression of previously-reported candidate marker genes related to cancer stem cells. (n=3, Dunnett's

test.). B) A comparison of OCT3/4, SOX2, KLF4, and NANOG expression. . Microarray probes for OCT3/4 (A_24_P144601) and KLF4 (A_23_P32233) are located in the coding regions, and the probes for OCT3/4 (A_23_P59138), SOX2 (A_33_P3361067) and NANOG (A_23_P204640) are in the 3' untranslated region. (n=3, *P<0.05, **P<0.01 Dunnett's test.).

Figure S5. The in situ hybridization of IL-6 in clinical samples (case 2, lesions 2, 3 in Table 1). A,B) Computed tomography (CT) images (A) and pictures of the surgical specimens (B) of a 67-year-old male patient with pT3N1M0 pm1 KRAS mutation-positive lung adenocarcinoma. Images of the primary lesion are shown on the left and pulmonary metastasis (PM) is shown on the right. (C)-(E) Immunohistochemical staining of E-Cadherin (left panels) and the in situ hybridization of IL-6 (right panels) in the intratumoral region of the primary lesion (C) and the PM lesion (D), and around the primary lesion (E) of the tumor. C) In this case, most of IL-6 mRNA-positive cells were E-cadherin-negative mesenchymal cells (arrowheads); however, a small number of IL6-mRNA-positive epithelial cancer cells were also detected (circle). D) IL-6 mRNA-positive lung epithelial cancer cells were also found in the PM lesion. E) The normal lung epithelial cells located immediately around the tumor were also IL-6 mRNA-positive (arrow).

Table S1. **The GO analysis of genes in the OSK-A549-Colony cells that were significantly upregulated in comparison to the parental A549 and OSK-A549-SN cells.** The genes were selected using a volcano plot. OSK-A549-Colony cells vs. OSK-A549-SN cells; volcano plot, P<0.03; Fold change, > 2.0; after the analysis of OSK-A549-Colony cells vs. the parental A549 cells; volcano plot, P<0.03; Fold change, > 2.0.

GO ID	GO ACCESSION	GO Term	p-value	corrected p-value	Count in selection	% Count in Selection	Count in total	% Count in total
947	GO:0001568	Blood vessel development	2.07E-07	7.40E-04	8	28.57143	426	2.395816
1259	GO:0001944	Vasculature development	2.94E-07	7.40E-04	8	28.57143	446	2.508295
4829	GO:0006690	Eicosanoid metabolic process	2.66E-07	7.40E-04	5	17.85714	90	0.506158
20244	GO:0043207	Response to external biotic stimulus	3.78E-07	7.40E-04	9	32.14286	644	3.621844
27087	GO:0051707|	Response to other organism	3.78E-07	7.40E-04	9	32.14286	644	3.621844
32341	GO:0072358	Cardiovascular system development	9.50E-08	7.40E-04	10	35.71429	738	4.150498
32342	GO:0072359	Circulatory system development	9.50E-08	7.40E-04	10	35.71429	738	4.150498
36724	GO:1901568	Fatty acid derivative metabolic process	2.66E-07	7.40E-04	5	17.85714	90	0.506158
5029	GO:0006950	Response to stress	5.08E-07	7.79E-04	17	60.71429	3167	17.81115
6863	GO:0009607	Response to biotic stimulus	5.47E-07	7.79E-04	9	32.14286	673	3.784939

Figure S1

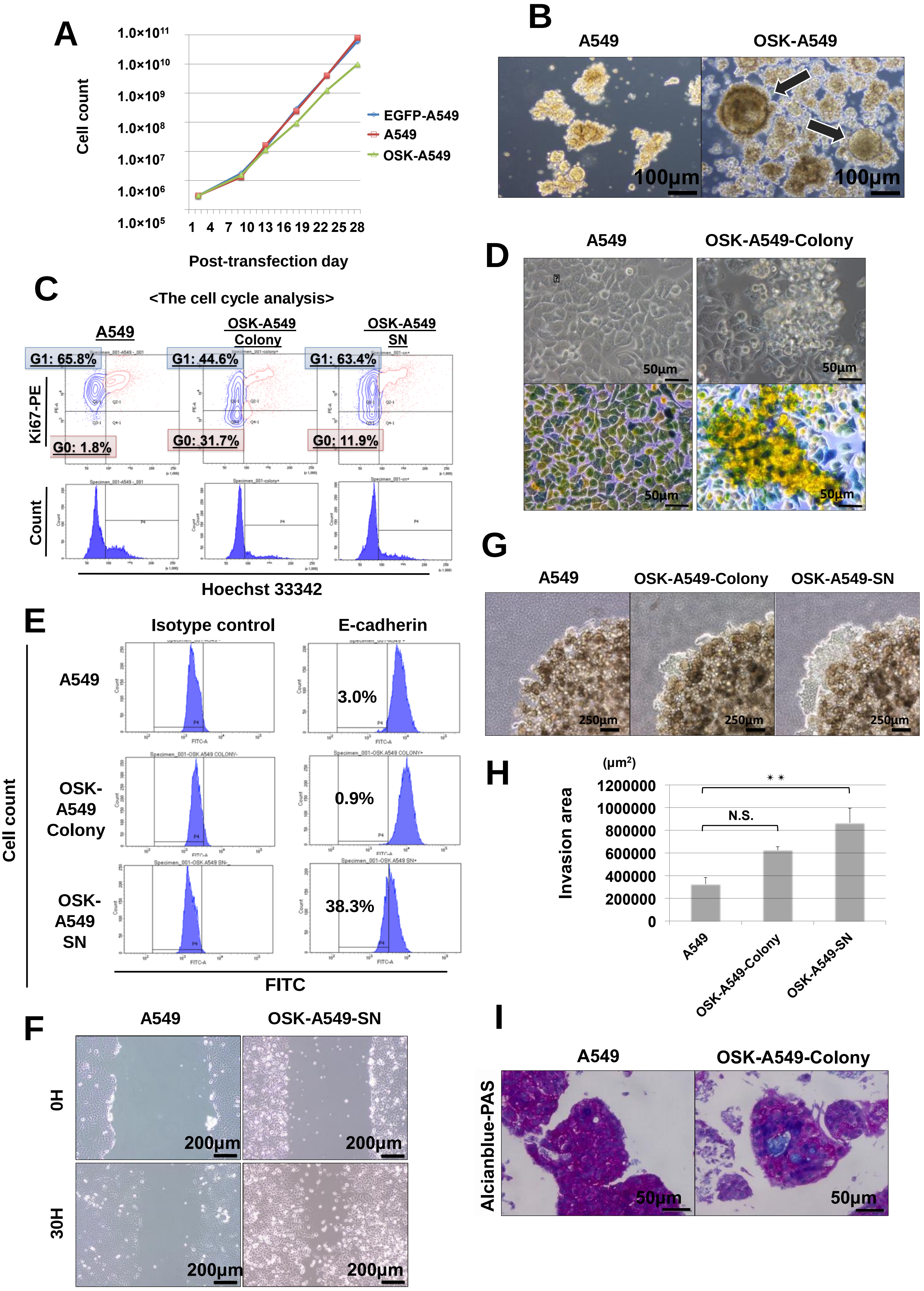

A
Cell count
1.0×10^11
1.0×10^10
1.0×10^9
1.0×10^8
1.0×10^7
1.0×10^6
1.0×10^5
1 4 7 10 13 16 19 22 25 28
Post-transfection day
EGFP-A549
A549
OSK-A549
B
A549
OSK-A549
100μm
100μm
C
<The cell cycle analysis>
A549
OSK-A549 Colony
OSK-A549 SN
G1: 65.8%
G1: 44.6%
G1: 63.4%
G0: 1.8%
G0: 31.7%
G0: 11.9%
Ki67-PE
Count
Hoechst 33342
D
A549
OSK-A549-Colony
50μm
E
Isotype control
E-cadherin
A549
OSK-A549 Colony
OSK-A549 SN
3.0%
0.9%
38.3%
Cell count
FITC
F
A549
OSK-A549-SN
0H
30H
200μm
G
A549
OSK-A549-Colony
OSK-A549-SN
250μm
H
(μm²)
Invasion area
1200000
1000000
800000
600000
400000
200000
0
＊＊
N.S.
A549
OSK-A549-Colony
OSK-A549-SN
I
A549
OSK-A549-Colony
Alcianblue-PAS
50μm

Figure S2

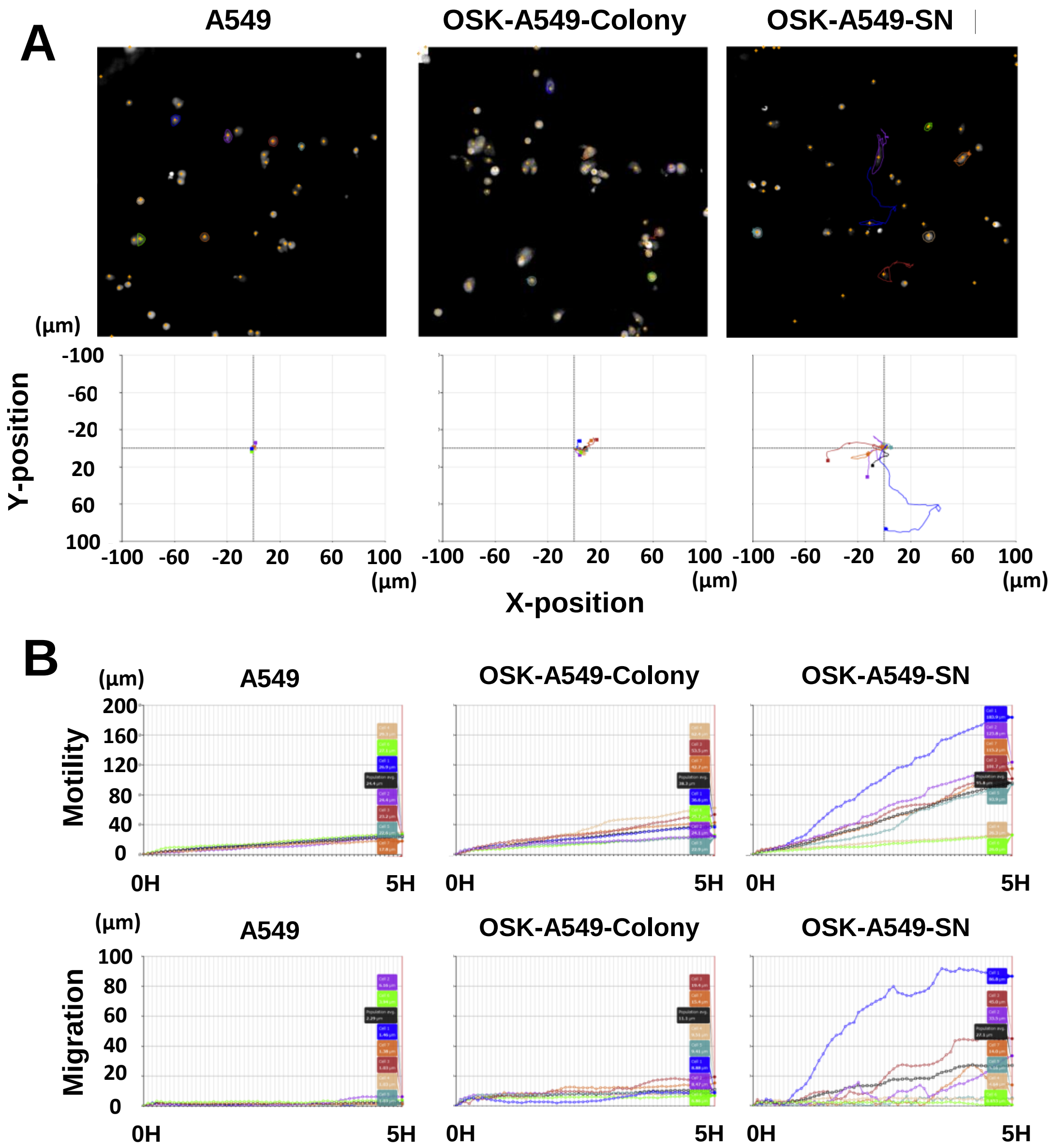
A
A549
OSK-A549-Colony
OSK-A549-SN
(µm)
Y-position
-100
-60
-20
20
60
100
X-position
(µm)
B
Motility
(µm)
200
160
120
80
40
0
0H
5H
Migration
100
80
60
40
20
0

Figure S3

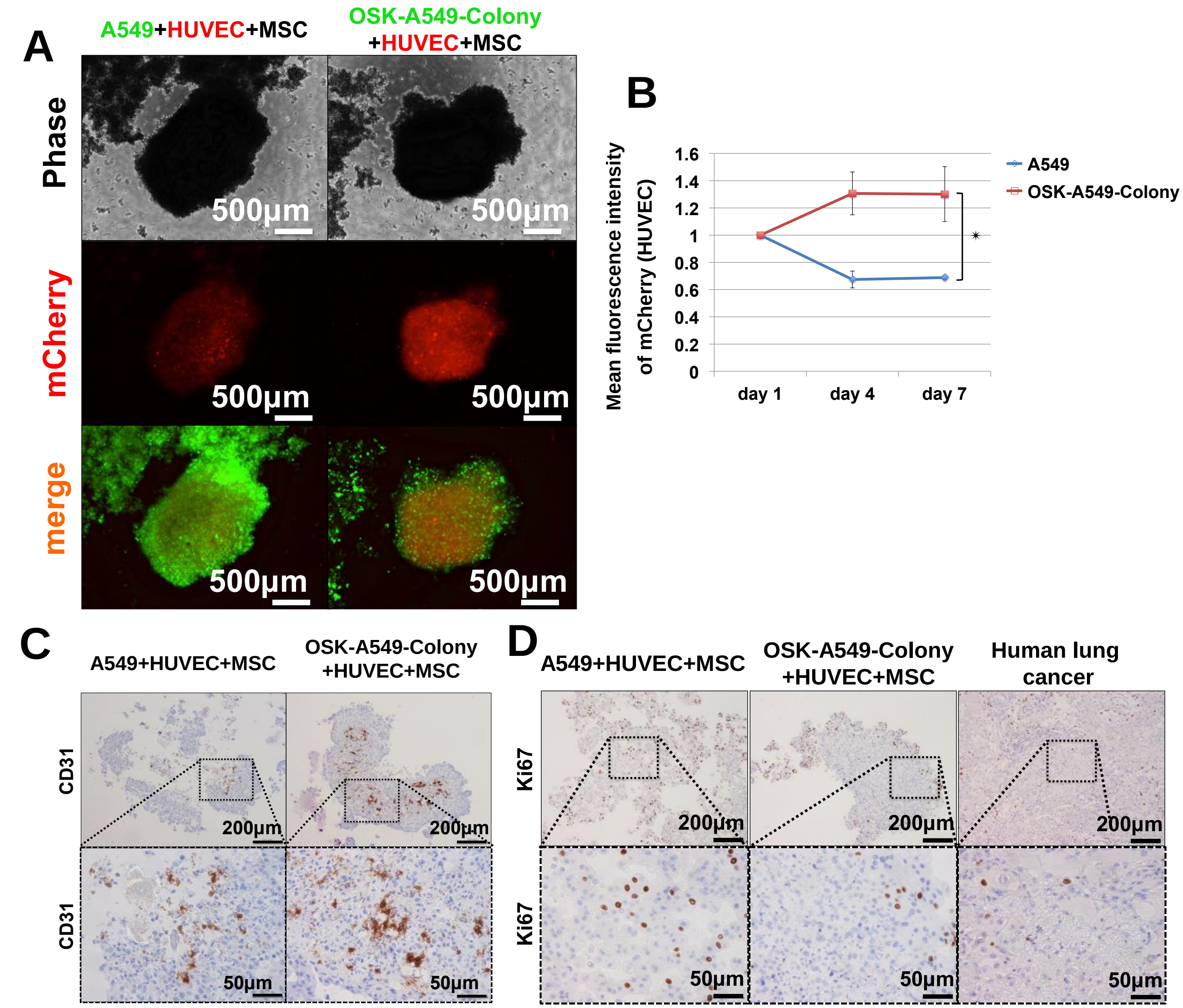

	A549+HUVEC+MSC	OSK-A549-Colony+HUVEC+MSC
Positive cells	34 (3.4%)	196 (18.0%)
Negative cells	986	1088

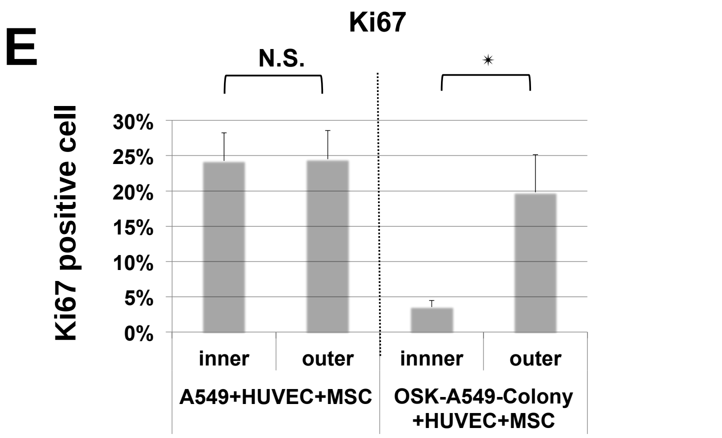

Figure S4

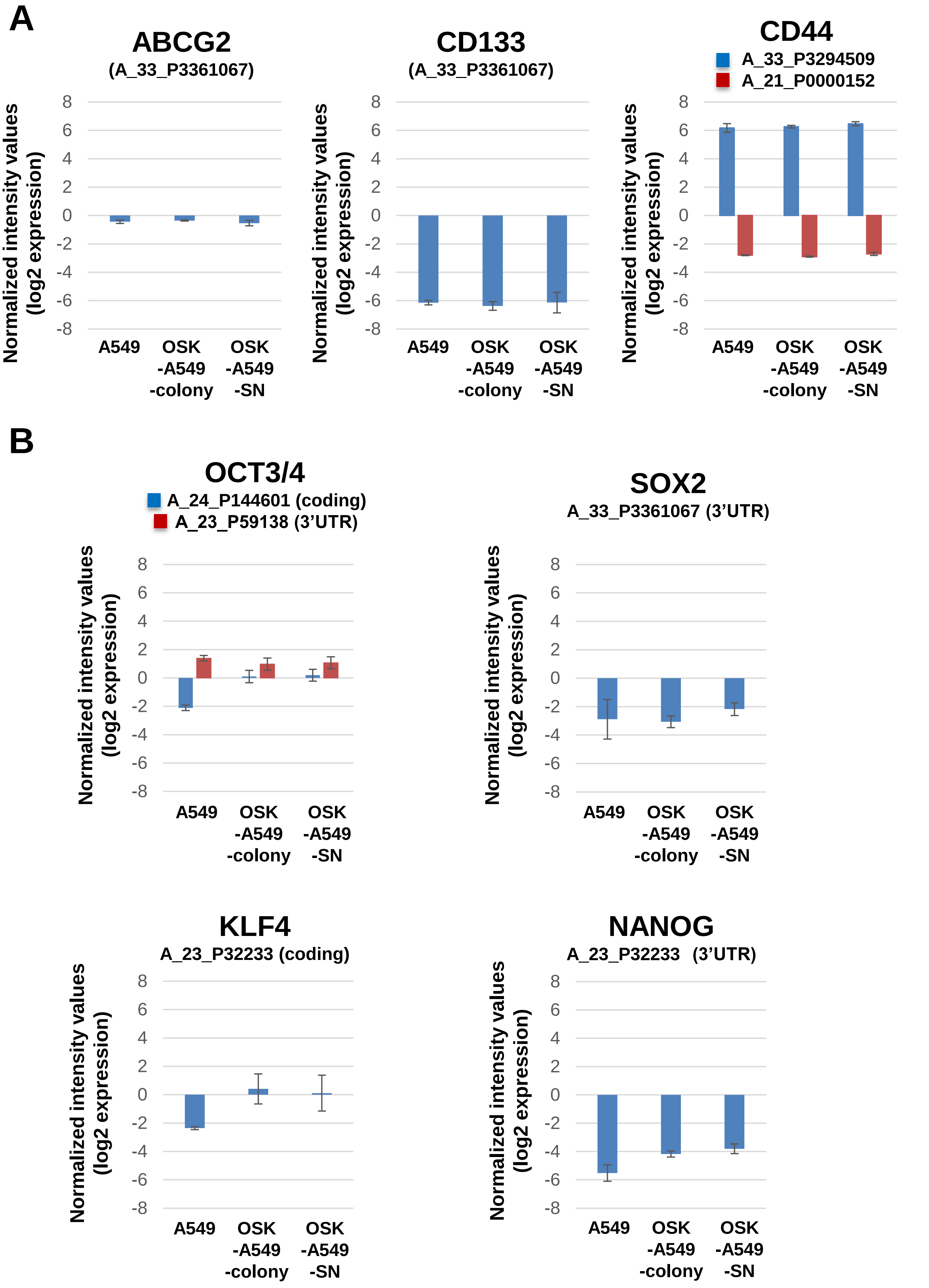
A
ABCG2
(A_33_P3361067)
CD133
(A_33_P3361067)
CD44
A_33_P3294509
A_21_P0000152
Normalized intensity values (log2 expression)
8
6
4
2
0
-2
-4
-6
-8
A549
OSK -A549 -colony
OSK -A549 -SN
B
OCT3/4
A_24_P144601 (coding)
A_23_P59138 (3'UTR)
SOX2
A_33_P3361067 (3'UTR)
KLF4
A_23_P32233 (coding)
NANOG
A_23_P32233 (3'UTR)

Figure S5

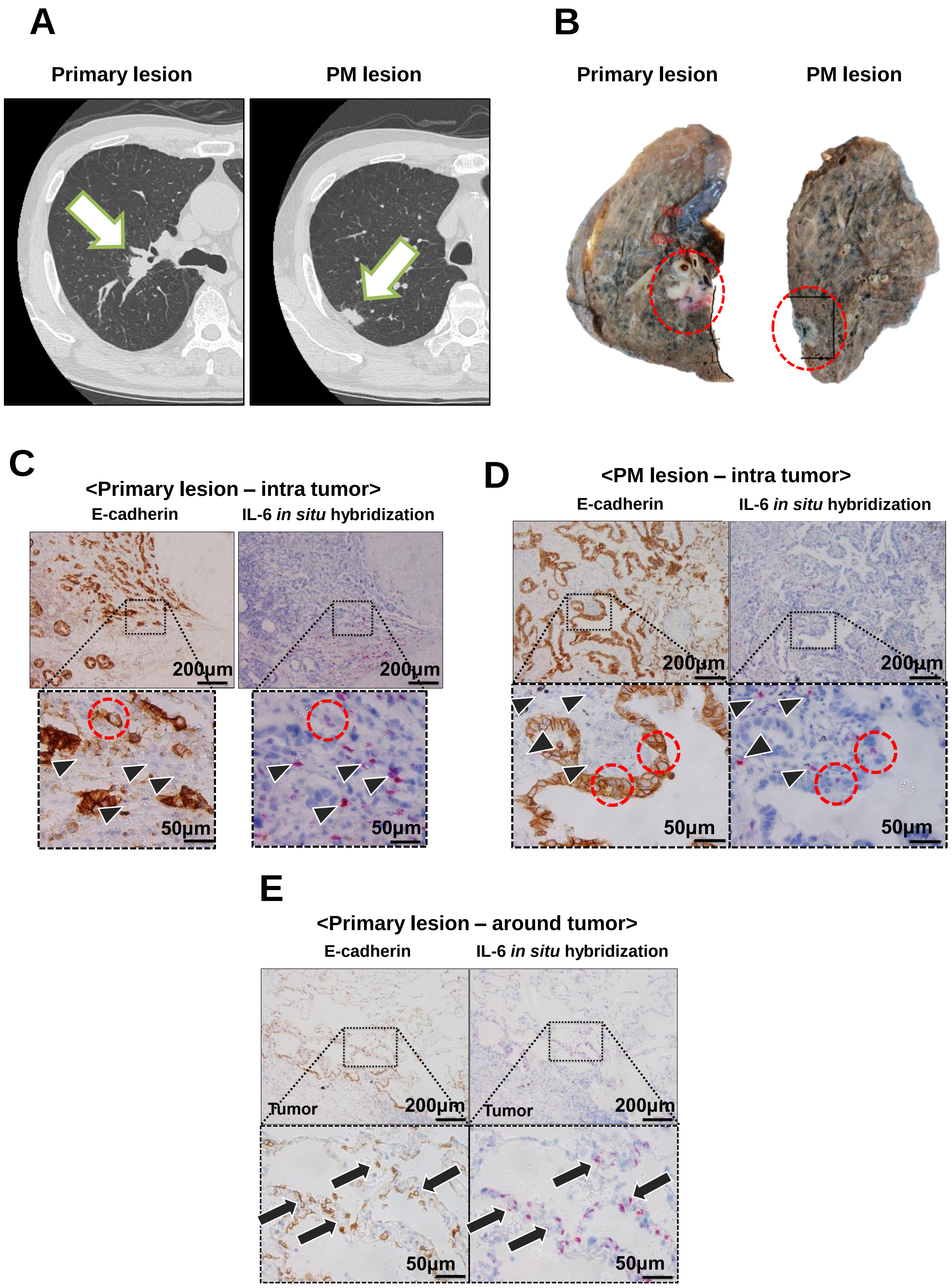
A
Primary lesion
PM lesion
B
Primary lesion
PM lesion
C
<Primary lesion – intra tumor>
E-cadherin
IL-6 *in situ* hybridization
200μm
200μm
50μm
50μm
D
<PM lesion – intra tumor>
E-cadherin
IL-6 *in situ* hybridization
200μm
200μm
50μm
50μm
E
<Primary lesion – around tumor>
E-cadherin
IL-6 *in situ* hybridization
Tumor
200μm
Tumor
200μm
50μm
50μm
